# A Homozygous *PPP1R21* Splice Variant Associated with Severe Developmental Delay, Absence of Speech, and Muscle Weakness Leads to Activated Proteasome Function

**DOI:** 10.1007/s12035-023-03219-9

**Published:** 2023-01-24

**Authors:** Andreas Hentschel, Nancy Meyer, Nicolai Kohlschmidt, Claudia Groß, Albert Sickmann, Ulrike Schara-Schmidt, Fabian Förster, Ana Töpf, Jon Christiansen, Rita Horvath, Matthias Vorgerd, Rachel Thompson, Kiran Polavarapu, Hanns Lochmüller, Corinna Preusse, Luis Hannappel, Anne Schänzer, Anika Grüneboom, Andrea Gangfuß, Andreas Roos

**Affiliations:** 1 Leibniz-Institut für Analytische Wissenschaften – ISAS – e.V, Dortmund, Germany; 2 Department of Pediatric Neurology, Centre for Neuromuscular Disorders, Centre for Translational Neuro- and Behavioral Sciences, University Duisburg-Essen, Essen, Germany; 3 Institute of Clinical Genetics and Tumor Genetics, Bonn, Germany; 4 The John Walton Muscular Dystrophy Research Centre, Translational and Clinical Research Institute, Newcastle University and Newcastle Hospitals NHS Foundation Trust, Newcastle Upon Tyne, UK; 5 Department of Clinical Neurosciences, John Van Geest Centre for Brain Repair, School of Clinical Medicine, University of Cambridge, Cambridge, UK; 6 Department of Neurology, Heimer Institute for Muscle Research, University Hospital Bergmannsheil, Ruhr University Bochum, Bochum, Germany; 7 Children’s Hospital of Eastern Ontario Research Institute; Division of Neurology, Department of Medicine, The Ottawa Hospital; and Brain and Mind Research Institute, University of Ottawa, Ottawa, Canada; 8 Department of Neuropathology, Charité - Universitätsmedizin Berlin, Berlin, Germany; 9 Institute of Neuropathology, Justus Liebig University, Gießen, Germany

**Keywords:** PPP1R21, NEDHFBA, Proteasome, Fibroblast cytoskeleton, Fibroblast proteomics, Fibroblast electron microscopy, Fibroblast filopodia

## Abstract

PPP1R21 acts as a co-factor for protein phosphatase 1 (PP1), an important serine/threonine phosphatase known to be essential for cell division, control of glycogen metabolism, protein synthesis, and muscle contractility. Bi-allelic pathogenic variants in *PPP1R21* were linked to a neurodevelopmental disorder with hypotonia, facial dysmorphism, and brain abnormalities (NEDHFBA) with pediatric onset. Functional studies unraveled impaired vesicular transport as being part of PPP1R21-related pathomechanism. To decipher further the pathophysiological processes leading to the clinical manifestation of NEDHFBA, we investigated the proteomic signature of fibroblasts derived from the first NEDHFBA patient harboring a splice-site mutation in *PPP1R21* and presenting with a milder phenotype. Proteomic findings and further functional studies demonstrate a profound activation of the ubiquitin–proteasome system with presence of protein aggregates and impact on cellular fitness and moreover suggest a cross-link between activation of the proteolytic system and cytoskeletal architecture (including filopodia) as exemplified on paradigmatic proteins including actin, thus extending the pathophysiological spectrum of the disease. In addition, the proteomic signature of *PPP1R21*-mutant fibroblasts displayed a dysregulation of a variety of proteins of neurological relevance. This includes increase proteins which might act toward antagonization of cellular stress burden in terms of pro-survival, a molecular finding which might accord with the presentation of a milder phenotype of our NEDHFBA patient.

**Supplementary Information:**

The online version contains supplementary material available at 10.1007/s12035-023-03219-9.

## Introduction

The protein phosphatase 1 (PP1) is a serine/threonine phosphatase that is known to be essential for cell division [1] and moreover important in the control of glycogen metabolism [2], protein synthesis, and muscle contractility despite many other processes. Furthermore, PP1 is known to be expressed during development in different neuronal cytoskeletal compartments [3], thus playing a crucial role in neural systems. One of the many known regulators of PP1 is the protein phosphatase 1 regulatory subunit 21 (PPP1R21) encoded by the *PPP1R21* gene, but until today, its exact molecular effect on PP1 remains elusive. Considering the crucial role of PP1 and its regulatory units, PPP1R21 is expected to also have an important effect in various cellular functions. Recent investigations describe a novel neurodevelopmental syndrome caused by bi-allelic *PPP1R21* loss-of-function variants and imply a role for PPP1R21 within the endosomal sorting process or endosome maturation pathway [4]. So far, 11 individuals with homozygous pathogenic variants in the *PPP1R21* gene have been linked to the neurological phenotype [4–8]. The phenotype of *PPP1R21* cases shows a broad spectrum of abnormalities. The majority of patients have a developmental delay/intellectual disability, coarse facial features with low set ears, muscle hypotonia, brain anomalies (e.g., reduced white matter and cerebellar vermis hypoplasia), feeding problems, ocular signs (strabismus/nystagmus), cardiac defects, and respiratory findings [7].

However, the underlying pathomechanism is still not fully understood. To address this lack of knowledge and to unravel pathophysiological processes related to the etiology of the *PPP1R21* disease, we utilized proteomics. This approach was prompted by the fact that proteomics was demonstrated to be a suitable technique to identify processes taking place in neurological diseases [9] and thus applied on fibroblasts derived from a patient carrying the first homozygous splice variant described for this gene. This approach indicated activation of the ubiquitin–proteasome system accompanied by dysregulation of cytoskeletal proteins, two findings not only verified by further comprehensive studies but also apparently functionally cross-linked in the pathophysiology of PPP1R21opathy. Further protein-dysregulations hint toward the activation of compensatory strategies, a molecular finding which would be consistent with the less severe phenotype seen in our patient.

## Materials and Methods

### Genetic Analysis

DNA samples from the proband and his parents were subjected to whole exome sequencing. Libraries were created with an Illumina exome capture (38 Mb target) kit and sequenced with a mean target coverage of 80x (Genomics Platform, Broad Institute of MIT and Harvard, Cambridge, USA). Data analysis was carried out in two stages, first searching for pathogenic/likely pathogenic variants in genes known to be associated with the clinical presentation (in silico panel). If no variants were identified, and given the known consanguinity in the family, the full exome was interrogated by applying a stringent criterion searching for homozygous highly damaging variants (i.e., nonsense, splice region, and frame-shift) absent in the control population (gnomAD; http://gnomad.broadinstitute.org).

### Sample Preparation for Proteomics

Cell lysis of cell pellets was performed by adding 200 µl of 50 mM Tris–HCl (pH 7.8) buffer, 1% SDS with cOmplete ULTRA protease inhibitor (Roche), and a sonication step with an ultrasound probe (30 s, 1 s/1 s, and amplitude 40%), followed by centrifugation at 4 °C and 20,000 g for 15 min. To precipitate the proteins, 3 ml of ice-cold acetone was added and incubated overnight at − 20 °C. After centrifugation at 4 °C for 20 min at 20,000 g, the supernatant was discarded, and the precipitated proteins were dried under a flow hood for a few minutes. Then, the dried protein pellet was dissolved by adding 8 M urea and mixing for one hour. After complete solubilization, the samples were diluted to 2 M urea using 10 mM ABC (ammonium bicarbonate, pH 7.8) buffer. To determine the protein concentration, a BCA (Pierce BCA protein assay kit) was performed according to the manufacturer's instructions. Reduction and carbamidomethylation of the samples were performed with 10 mM TCEP (tris-(2-carboxyethyl)-phosphine) for an incubation time of 30 min at 37 °C and with 15 mM IAA (iodoacetamide) for an incubation time of 30 min at RT, respectively. Proteins were hydrolyzed in solution with a trypsin to protein ratio of 1:100. Tryptic digestion was performed overnight at 37 °C and stopped the next day by addition of 99% formic acid (FA). Samples were desalted by solid phase extraction using C18 filter cartridges (Waters) by washing with 0.1% trifluoroacetic acid (TFA). This was followed by elution with 80% acetonitrile (ACN). The purified samples were dried using a vacuum concentrator. The concentration was adjusted to 1 µg/µl with 0.1% TFA.

All proteolytic digests were checked for complete hydrolysation using a monolithic column separation (PepSwift monolithic PS-DVB PL-CAP200-PM, Dionex) on an inert Ultimate 3000 HPLC (Dionex) by direct injection of 1 μg sample. A binary gradient (solvent A: 0.1% TFA, solvent B: 0.08% TFA, 84% ACN) of 5–12% B in 5 min and then of 12–50% B in 15 min was applied at a flow rate of 2.2 μl/min and at 60 °C. UV traces were recorded at 214 nm [10].

### Mass Spectrometry Analysis

Mass spectrometric measurements were performed in data-independent acquisition (DIA) mode. For this purpose, each sample to be analyzed was mixed with an appropriate amount of iRT standard peptides (Biognosys). A total of 1 µg of each sample was used for analysis. The final hydrolysate was loaded onto an Ultimate 3000 Rapid Separation Liquid Chromatography (RSLC) nanosystem with a ProFlow flow control in line with a Fusion Lumos Tribrid mass spectrometer (both from Thermo Scientific). After initial loading, peptides were pre-concentrated on a trapping column (Acclaim C18 PepMap100, 100 µm, 2 cm) using 0.1% TFA at a flow rate of 10 µl/min. Subsequent sample separation was performed on a reversed-phase column (Acclaim C18 PepMap100, 75 µm, 50 cm) using a binary gradient: 3% solvent B (84% ACN with 0.1% TFA) for 10 min, a linear increase in solvent B to 35% for 120 min, and a linear increase in solvent B to 95% for 10 min followed by a linear reduction in solvent B to 3% for 5 min. Full MS scans were acquired from 300 to 1100 m/z at a resolution of 60,000 (Orbitrap) using the polysiloxane ion at 445.12002 m/z as the lock mass. The automatic gain control (AGC) was set to 5E5 and the maximum injection time to 20 ms. The full MS scans were followed by 30 DIA windows acquired at a resolution of 30,000 (Orbitrap) with AGC set to 1E6, a maximum injection time of 60 ms, and a normalized collision energy of 32 (HCD).

### Data Analysis

The acquired data were imported into Spectronaut software (Biognosys). The human proteome data from UniProt (www.uniprot.org), containing 20,374 entries, were selected as the proteome background. Processing settings were as follows: enzyme was trypsin, minimum and maximum peptide length were set to 7 and 52, respectively, and missed cleavages were set to 2. Carbamidomethyl for cysteine was set as a fixed modification. Acetyl (protein N-term) and oxidation of methionine were set as variable modifications. All settings related to library generation including tolerances, identification, filters, iRT calibration, and workflow were left at factory defaults. For relative quantification, the Top N max 3 option was chosen, meaning that for each protein the average of the 3 peptides identified with the highest intensity was taken to calculate the quantitative value for that protein. The analyzed data were matched against an in-house spectral library [11] and protein identification was performed using the pulsar search engine included in Spectronaut.

### Visualization of Regulated Proteins and GO Terms

Voronoi diagrams depicting functional categories and proteins were generated using the online software Proteomaps [12]. The size of a protein tile depends on the size of the calculated ratio. This was determined as the mean of three biological replicates.

### Protein Network Analysis

To investigate possible functional protein interactions in silico, the resulting list of dysregulated proteins was entered into the STRING database [13]. For this purpose, the significance score for the interactions in the STRING network was calculated by combining the categories of co-expression, experimental, knowledge, and text mining from the database. The interaction score was set to 0.4. A score of 0.5 would mean that every second interaction could be erroneous or false positive. The smaller this value is chosen, the more trustworthy the displayed interactions are.

### Electron Microscopy on Fibroblasts

To investigate the presence of protein aggregates on the ultra-structural level, transmission electron microscopy (TEM) was carried out: cell pellets of PPP1R21-mutant and two controls fibroblasts were fixed with 2.9% glutaraldehyde/0.4 M phosphate buffered saline (PBS) and were processed with a Leica EM TP tissue processor with 1%-osmium-tetroxide and embedded in resin. For electron microscopy, ultrathin sections were contrasted with 3% lead citrate trihydrate with a Leica EM AC20 (Ultrastain kit II) and were examined using a Zeiss EM 109 transmission electron microscope equipped with a Slowscan-2 K-CCD-digital camera (2 K-wide-angle Sharp:eye).

Myelin figures (MF) were analyzed at ultrastructural images (magnification × 4.400 and 12.000). MF were counted in all fibroblasts in which the nucleus was gated.

### Investigation of Proteasome Activity and Cellular Viability

The activity of the proteasome was measured in cultured fibroblasts derived from the *PPP1R21*-patient and two controls (grouped for further data analysis) utilizing the Proteasome Activity Assay Kit (Abcam ab107921) according to the manufacturer's instructions. To block proteasomal activity, cells were treated with 10 µM MG132 for 16 h. Cellular viability was monitored by measuring the metabolic activity of fibroblasts making use of an MTT-assay (a colorimetric assay; 3-[4,5-dimethylthiazol-2-yl]-2,5 diphenyl tetrazolium bromide). The MTT assay way carried out as described previously [14].

### Immunoblot Studies on Whole Protein Extracts of Human Fibroblasts

Immunoblot studies on whole proteins extracts of the PPP1R21-patient and two pooled controls were carried out as described previously [15] utilizing the primary antibodies listed in supplementary Table 2.

### Transcript Studies on cDNA of Human Fibroblasts

Total RNA was extracted from fibroblasts of the patient, as well as controls, using a Trizol-chloroform method and reversely transcribed into cDNA using the High-Capacity cDNA Archive Kit (Applied Biosystems, Foster City, CA). For qPCR reactions, 10 ng of cDNA was used for analysis on the QuantStudio 6 Flex System (Applied Biosystems, Foster City, CA) with the following running conditions: 95 °C 0:20, 95 °C 0:01, 60 °C 0:20, and 45 cycles (values above 40 cycles were defined as “not expressed”). All targeted transcripts were run as triplicates. The TaqMan® Gene Exp Assay (Life Technologies/ ThermoFisher) is listed as follows: *ATF4* Hs00909569_g1, *ATP6* Hs00232586_m1, *BAG3* HS00188713_m1, *BECN1* Hs00186838_m1, *CALR*Hs00376764_m1, *CALU* Hs04992290_s1, *CANX* Hs01558409_m1, *EDEM1* Hs00976004_m1, *EIF2A* Hs00230684_m1, *EIF2AK3/PERK* Hs00984003_m1, *ERN1/IRE1* Hs01086607_m1, *GRP170/HYOU1* Hs00197328_m1, *HSPA1B* Hs01040501_sH, *HSPA5/GRP78/BIP* Hs00607129_gH, *HSPA8* Hs03044880_gH, *HSPB8* Hs00205056_m1, *HSP90B1/GRP94* Hs00427665_g1, *LAMP1* Hs00931461_m1, *LC3 / MAP1* Hs01076567_g1, *LMP7/PSMB8* Hs00544760_g1, *PDIA2* Hs00429010_m1, *PDIA3* Hs00607126_m1, *PGK1* Hs99999906_m1 (reference gene), *PSME1* Hs00389210_g1, *PSME2* Hs01923165_u1, *SEC63* Hs00273093_m1, and *XBP1* Hs00231936_m1.

Gene expression of patient fibroblast was determined three times and illustrated by the fold change compared to the mean level of control fibroblasts. A *p* value < 0.05 was considered significant and calculated with GraphPad Prism 9.2.0 (GraphPad Software, Inc., La Jolla, CA, USA).

### Confocal Laser Scanning Microscopy (CLSM) and Image Processing of Fluorescent-Labeled Human Fibroblasts

High resolution scans of human fibroblasts stained with FITC-phalloidin were performed using a Leica TCS SP8 confocal laser scanning microscope with acousto-optic tunable filters, an acousto-optical beam splitter, internal hybrid detectors (HyD SP), and a LMT200 high precision scanning stage. Imaging of coverslip-embedded samples was performed via a Leica HC PL APO 63x/1.20 W CORR objective in combination with variable digital zoom factors according to fibroblast size. Phalloidin-AlexaFluor488 labeled actin fibers were excited with an argon laser at 488 nm and detected with an internal HyD at 500–550 nm. In a second sequence, DAPI was excited with a 405 nm diode-pumped solid-state laser and detected by an internal HyD at 450–500 nm. Generated images were deconvoluted with Huygens Professional (SVI) and reconstructed with Imaris software (Bitplane). To generate the circular color map and the coherency orientation index (COI), the confocal scanned z-stacks were loaded into ImageJ and converted into maximum intensity projections (MIP) using the Z Project tool. Single cells were identified by manual region of interest (ROI) definition and optically separated by clearing the area outside the ROI. Afterwards, the isolated cells were horizontally aligned, the color maps were generated, and the COI of the respective cells was determined with the help of the OrientationJ plugin. Statistical analysis of the COI values was performed with GraphPad Prism 9.

The filopodia of human fibroblasts were manually isolated optically from the rest of the actin-fiber skeleton of the cell body based on their morphological characteristics and quantified accordingly. The length as well as the branching of filopodia was quantified using the ImageJ plugin "Analyze Skeleton (2D/3D)" [16].

## Results

### Clinical Findings

The patient, a 6-year-old boy, is the first child of healthy consanguineous parents of Turkish origin. Pregnancy was uneventful, mother being 24 years old at conception. Birth was an uncomplicated with spontaneous delivery at 38 + 1 weeks of gestation (Apgar score 10/10/10, umbilical cord pH 7.28). Birth measures were as following: weight = 3640 g (76. percentile, 0.7z), length = 53 cm (78. percentile, 0.78z), and head circumference = 33,5 cm (15. percentile, − 1.05z) (*z*-scores and percentiles: [17]).

At the age of 2 h, the patient was admitted to the neonatal care unit due to a cardiopulmonary adaption disorder. He needed respiratory support via high flow nasal cannula (HFNC) and intravenous fluids for two days. Directly postnatally, an intermittent strabismus of both eyes, a slight mandibular retrognathia, a slight thoracic instability, and low-set ears were noticed. Muscle tone was truncal hypertonic and peripheral hypotonic. Magnetic resonance imaging (MRI) of the head showed no malformations (small bleeding foci in the right central region), electroencephalography (EEG), and echocardiography showed normal results. Visually evoked potentials were abnormal but ophthalmologic examination showed normal results.

At 7 months of age, the parents reported good thriving. In the clinical examination, the child showed a generalized hypotonia. In the traction test, the head was taken along only briefly, in prone position also only brief head control was possible. No weight assumption in held stance. Social smile, vocalization, and normal interaction with his mother were noticed. Strabismus convergens alternans was still present.

At the age of one year, the parents reported a developmental delay. He had not yet learned to crawl, sitting without support was not possible. He could roll from prone to supine. The MRI follow-up showed a moderate periventricular leukomalacia (PVL).

At age four years, the boy attempted to communicate through sounds and vocalizations, no speech production. Social smile was shown. The skull was asymmetrical (plagiocephaly), the face broad. Pupillary reaction to light was regular on both sides, he followed a light source. During sleep, the eyelids were half open. The bridge of the nose was narrow, as were the lips; the philtrum was long. The ears were large and low set, slightly asymmetrical. The muscle tone was markedly hypotonic. No raising of arms, no grasping was shown. He was able to turn from supine to prone position. Build was strong, trunk to limb ratio proportionate. Skin was fair, no pigment abnormalities present, the patient had dark blond hair. The hands showed a single transverse palmar crease. Chest was flat, heart and lungs auscultation were unremarkable, no liver or spleen enlargement existing. Genitals were male, prepubertal, right testis was descended, left not palpable (cryptorchidism). Body weight 20 kg (90th percentile, 1.26z), body height 104 cm (49th percentile, − 0.02z), BMI 18.5 kg/m^2^ (96th percentile, 1.74z) (*z*-scores and percentiles: [18]), and head circumference 50.5 cm (30th percentile, − 0.53z) (*z*-score and percentile: [19]).

Based on the lack of the full clinical presentation of NEDHFBA known from the literature (MIM: 619,383), especially regarding cardiac abnormalities, scoliosis, and CNS anomalies, we postulate the presence of a milder phenotype in our patient.

### Molecular Genetic Findings

**Fig. 1:**
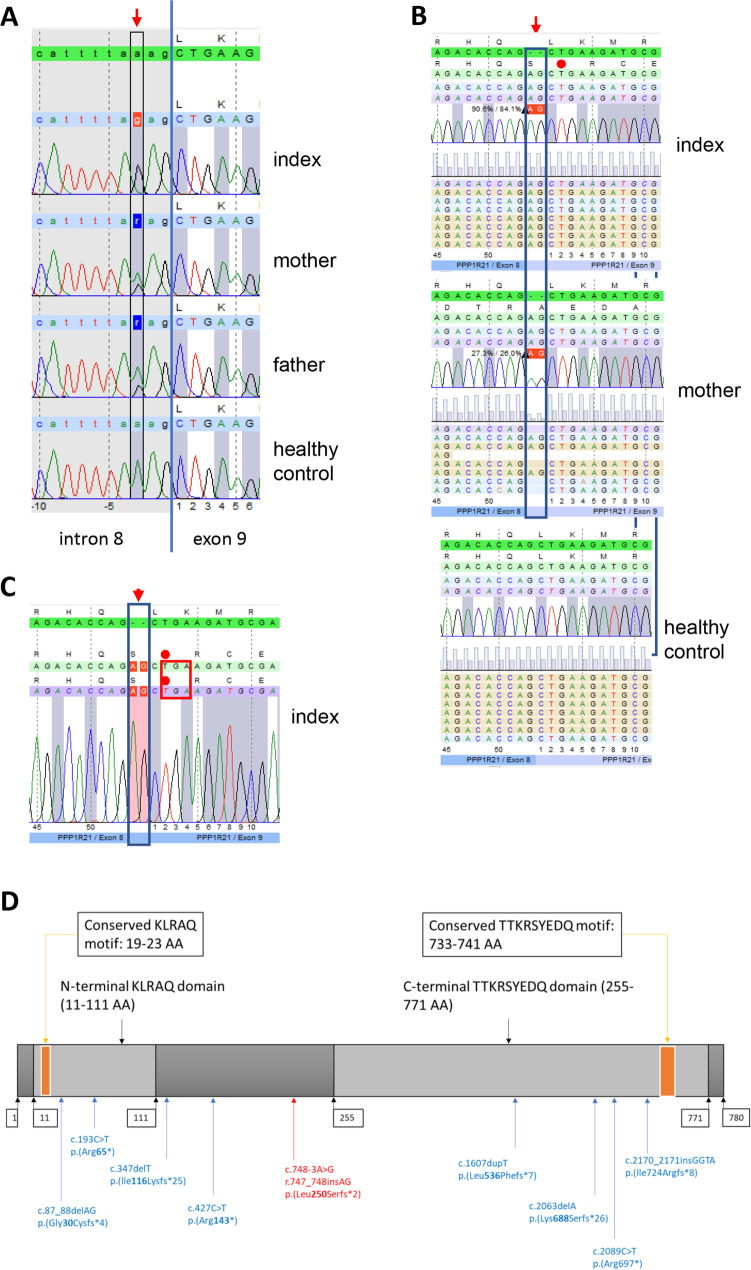
Molecular genetic findings. **A** Sanger sequencing of genomic DNA extracted from peripheral blood confirmed the splice site variant at position − 3 of intron 8 of the *PPP1R21* gene (c.748-3A > G) presenting as homozygous in the index patient and heterozygous in both parents, respectively. A wild-type sequence of a healthy control individual is shown in the lower panel. **B** Next generation sequencing of *PPP1R21* cDNA obtained from RNA isolated from peripheral blood samples of the index patient, the mother, and a healthy control; utilization of an alternative acceptor site leads to an insertion of two nucleotides (r.747_748insAG) between exons 8 and 9 of the resulting mRNA is seen in the index patient and his mother. Due to this insertion, the frameshift results in a premature stop codon at the beginning of exon 9 (p.Leu250Serfs*2). **C** Sanger sequencing of cDNA obtained from RNA isolated from cultured fibroblasts of the index patient confirms the insertion of two nucleotides between the exons 8 and 9 resulting in a frame shift and a premature stop codon at the beginning of exon 9 (p.Leu250Serfs*2). **D** Illustration of PPP1R21 structure including labeling of mutation site(s) on both, the nucleic acid and the amino acid (AA) level. Mutations reported previously (see references [4, 7, 8]) are labeled in blue whereas the pathogenic variant identified in our patient is labeled in red. Numbers in boxes refer to amino acid positions

The full exome was interrogated by applying a stringent criterion searching for homozygous highly damaging variants (i.e., nonsense, splice region, and frame-shift) absent in the control population (gnomAD; http://gnomad.broadinstitute.org). Thus, a homozygous splice region variant in the *PPP1R21* gene was identified (hg19:2:48692629A > G; c.748-3A > G). As expected, unaffected parents were heterozygous for the variant. Next generation sequencing results were confirmed by Sanger sequencing of an amplicon spanning the intron 8 and exon 9 junction of *PPP1R21* (Fig. 1A). Furthermore, the effect of this splice site variant was investigated at the cDNA level based on RNA extracted from peripheral blood showing that this homozygous variant leads to the insertion of two nucleotides on the mRNA (r.747_748insAG) in turn leading to a frameshift mutation at the beginning of exon 9 (p.Leu250Serfs*2) (Fig. 1B). The same finding was obtained when analyzing cDNA obtained from cultured fibroblasts from the patient (Fig. 1C).

### PPP21R1 Expression in Human Fibroblasts

**Fig. 2:**
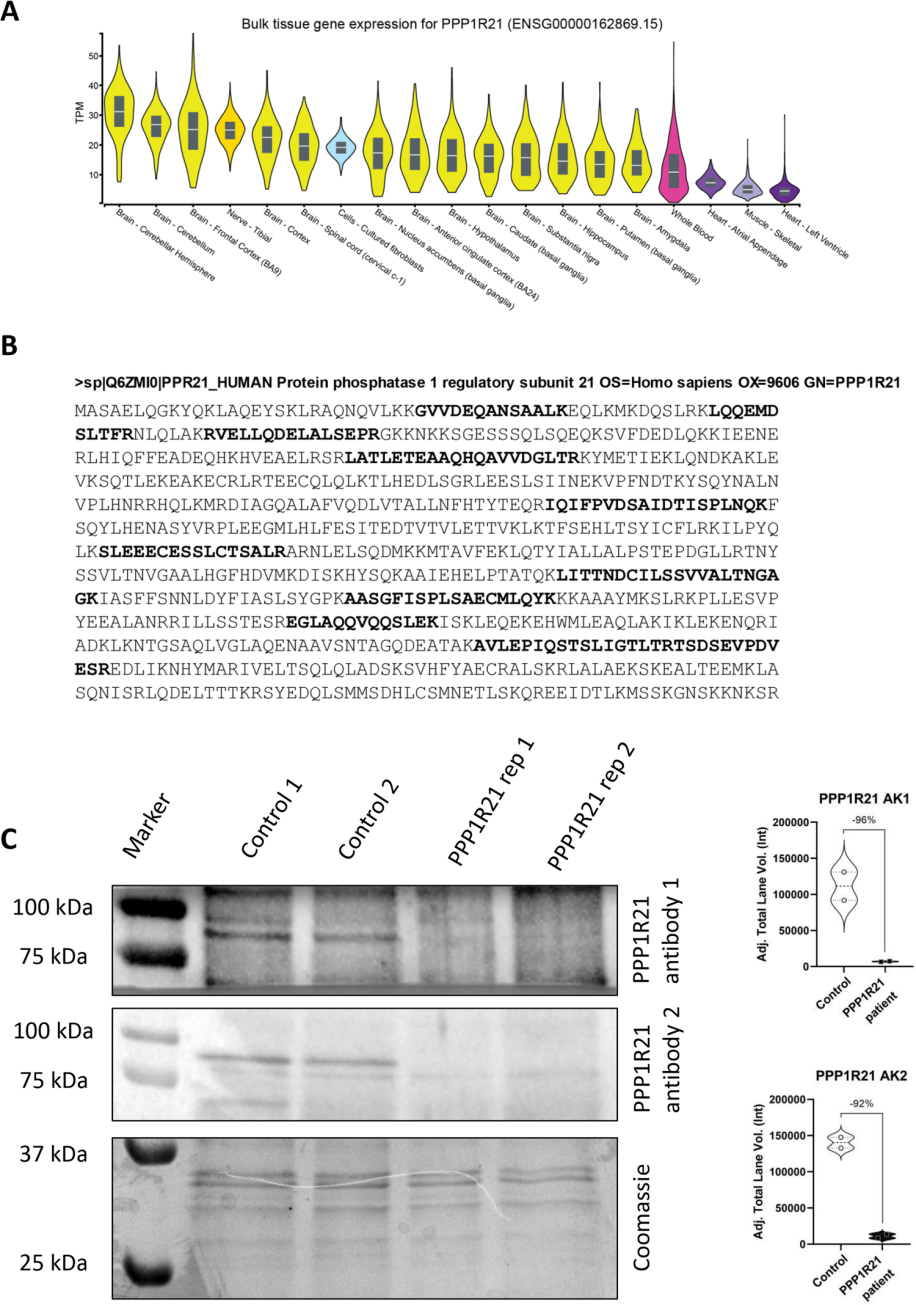
PPP1R21 expression in human fibroblasts. **A** GTEx-based in silico analysis of *PPP1R21* revealed expression in nervous tissues (yellow), cultured fibroblasts (blue), blood (pink), skeletal (light purple), and cardiac muscle (dark purple). **B** Sequence coverage of PPP1R21 based on unique peptides catalog in our spectral library. **C** Immunoblot studies (including quantification) utilizing two anti-PPP1R21 antibodies on whole protein extracts show loss of PPP1R21 protein abundance in patient-derived fibroblasts compared to controls. Coomassie staining is shown to show equal protein loading

A GTex-based in silico analysis was carried out and showed presence of *PPP1R21* transcripts in fibroblasts in addition to nervous and muscle tissue (Fig. 2A). By screening our in-house spectral library [20], we identified PPP1R21 with 12 unique peptides in total, covering 26.25% across the whole protein sequence (Fig. 2B). These combined findings support fibroblasts as a suitable in vitro model to study pathobiochemical processes taking place in case of deficiency of functional PPP1R21. To prove pathogenicity of the identified homozygous splice site variant leading to a pre-mature stop codon at the beginning of exon 9 in *PPP1R21*, we next analyzed PPP1R21 protein levels by immunoblotting. Utilizing two different antibodies (two different epitopes), we found complete loss of protein expression on protein extracts from patient fibroblasts compared to matched controls (Fig. 2C).

### Proteomic Signature of *PPP1R21* Patient-Derived Fibroblasts

**Fig. 3:**
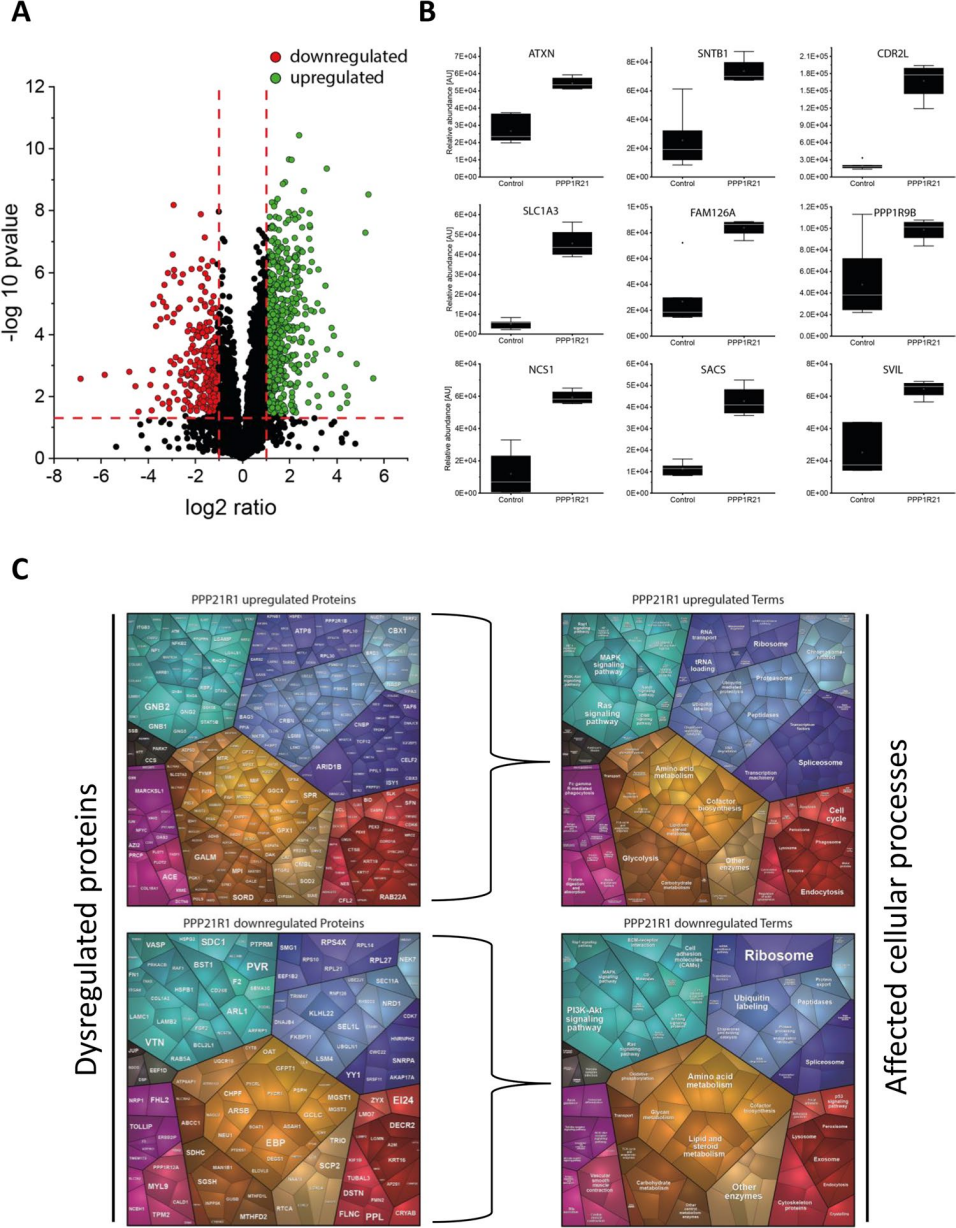
Proteomic profiling on *PPP1R21* patient fibroblasts. **A** Volcano plot highlighting statistically significant increased proteins (green dots) as well as decreased proteins (red dots). **B** Box plots of selected increased proteins including ATXN (ataxin-10), SNTB1 (Beta-1-synthrophin), CDR2L (cerebellar degeneration-related protein 2-like), SLC1A3 (excitatory amino acid transporter 1), FAM126A (hyccin), PPP1R9B (neurabin-2), NCS1 (neuronal calcium sensor 1), SACS (sacsin), and SVIL (supervillin) to visualize affection of neuronal and neuromuscular relevant proteins based on loss of functional PPP1R21 in cultured human fibroblasts. **C** Proteomaps-based analysis to decipher the impact on dysregulated proteins (left panel) on diverse biological and cellular processes (right panel). This analysis was performed for upregulated (upper panel) and downregulated (lower panel) proteins, respectively

By utilizing LC–MS/MS for proteomic profiling and taking advantage of our in-house spectral library (see Materials and Methods), we identified 4801 proteins in the *PPP1R21*-patient derived samples, of which 4701 were represented with at least two unique peptides. Focusing on these 4701 proteins for further data analysis, 853 (18.15%) proteins were significantly dysregulated with a *p* value of < 0.05 with 584 proteins being up- and 269 proteins being downregulated, respectively (Fig. 3A).

Our proteomic data revealed an increase of proteins belonging to the ubiquitin–proteasome system including PSMB9, DTX3L, TRIM16, PSMG4, CRBN, TTC3, COMMD8, UBE2L6, PSME2, PSMD10, UBE2Z, PSMB10, AGAP3, RPS27A, UBE20, TRIM21, HECTD3, TMUB1, OBSL1, UBE2N, PSMG2, OS9, UBE2L3, CLU, UBXN7, USP7, SGTA, PSMA5, USP24, CUL7, BABAM1, and PSMD7 (Suppl. Tab. 1), thus indicating an activation of this multi-catalytical protein clearance machinery.

Among all upregulated proteins, the excitatory amino acid transporter 1 (P43003; ninefold increase), the cerebellar degeneration-related protein 2-like (Q86X02; sevenfold increase), the neuronal calcium sensor 1 (P62166; fivefold increased), and sacsin (Q9NZJ4; 3.8-fold increase) are particularly interesting, as they are directly related to the neuronal system. Further upregulated proteins with known roles in neuronal or neuromuscular function and maintenance being include supervillin (SVIL; O95425), hyccin (Q9BYI3), beta-1-synthropin (Q13884), neurabin-2 (PPP1R9B; Q96SB3), and ataxin-10 (ATXN; Q9UBB4) (Fig. 3B).

Concerning downregulated proteins, tropomyosin alpha-1 chain (P09493; 25-fold decrease), filamin C (Q14315; 2.5-fold decrease), and tropomyosin (P07951; 2.5-fold decrease) are notable. These proteins are crucial for intracellular integrity and stability as well as for muscle contraction, myogenesis, and maintenance of structural integrity of muscle fibers. Furthermore, tenascin-X (P22105) and fibulin-5 (Q9UBX5) show a 12.5-fold and a 6.6-fold downregulation, respectively. These proteins are responsible for structural assembly and stability by strengthening the binding between cells and with the extracellular matrix. Furthermore, we observed a profound decrease of proteins responsible for growth and formation of neural structures such as neurite extension, axon growth, and formation of neuronal circuits. These proteins include CD166 (Q13740; 5.9-fold decrease), glia-derived-nexin (P07093; fivefold decrease), and neuropilin1 (O14786; 3.3-fold decrease) (Suppl. Tab. 1). All functional and disease-related information were obtained from “uniport” (www.uniprot.org). The proteomic profiling data have been deposited in the ProteomeXchange Consortium via the PRIDE partner repository with the dataset identifier PXD034993 and a list of all regulated proteins can be found in the supplement (Suppl. Tab. 1).

#### Cellular Processes Influenced by Proteins with Increased Abundance

Our proteomaps-based in silico analysis of cellular functions influenced by upregulated proteins revealed an impact on metabolic processes (including glycolysis and amino acid metabolism), on signaling pathways (including RAS and MAPK signaling), on RNA-processing (including splicing and translation), and on endocytosis and protein clearance (including proteasome). An impact on cell cycle and apoptosis is also indicated (Fig. 3C).

#### Cellular Processes Influenced by Proteins with Decreased Abundance

Our proteomaps-based in silico analysis of cellular functions influenced by down-regulated proteins revealed an impact on metabolic processes (including lipid, steroid, and carbohydrate metabolism), on cytoskeleton, on PI3K-AKT signaling as well as on ribosomal function (Fig. 3C).

### Proteasomal Activity in *PPP1R21* Patient-Derived Fibroblasts

**Fig. 4:**
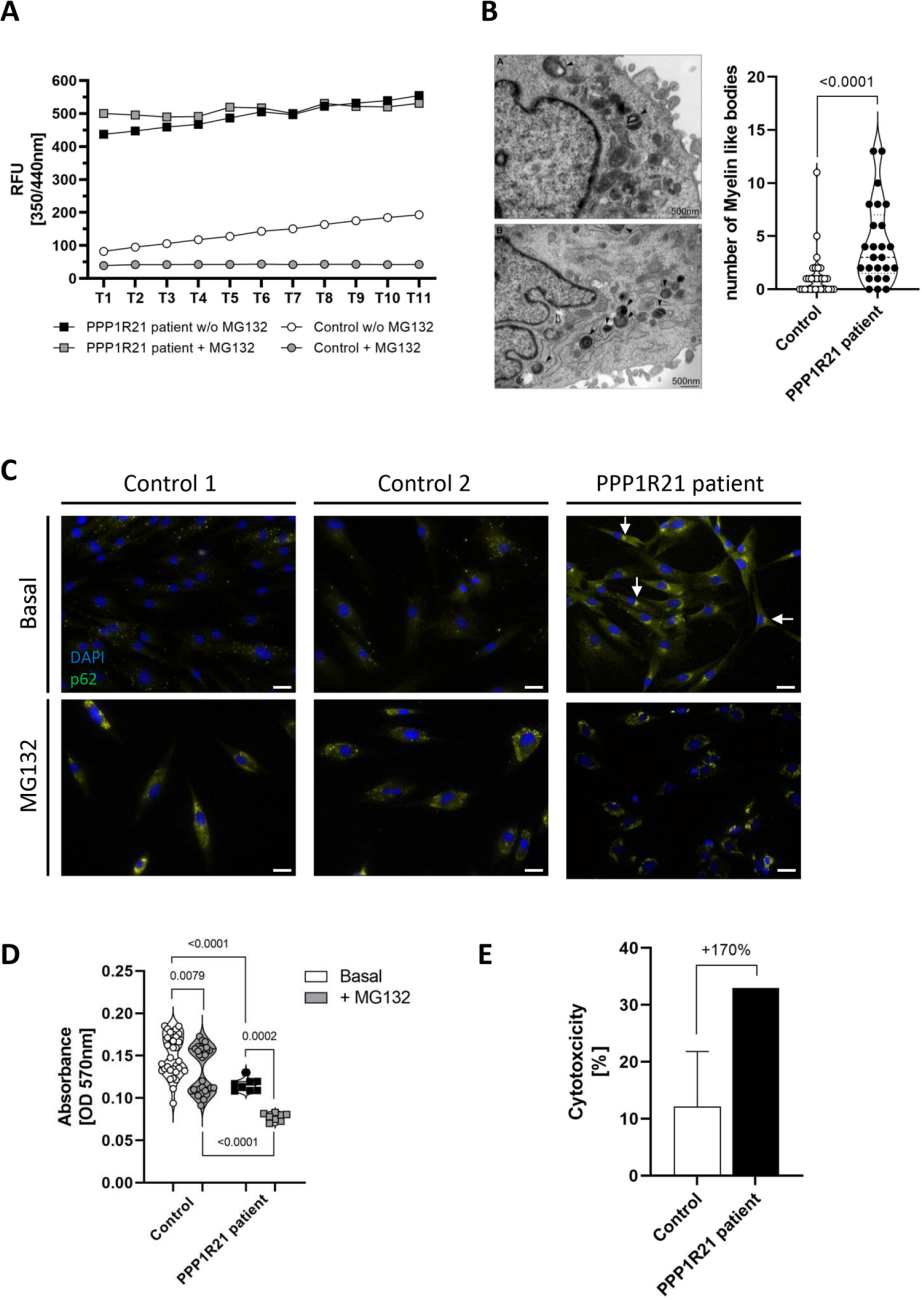
Cellular studies on the functional and microscopic level. **A** Investigation of proteasomal activity shows a five-fold increase of proteasomal function in *PPP1R21* patient-derived fibroblasts compared to pooled controls under basal conditions. After treatment with MG132 (10 µM for 16 h), control cells do not show proteasomal activity whereas there is no effect of treatment in fibroblasts lacking PPP1R21. **B** Ultrastructural analysis of cultured fibroblasts; only single myelin figures are present in the cytoplasm of control fibroblast as exemplified in the patient-derived (upper panel) and control cells (lower panel). In comparison to two controls analyzed, in *PPP1R21*-mutant fibroblast number of myelin-like bodies is increased as shown by counting respective numbers presented in the box plots (right panel). **C** Immunofluorescence studies on *PPP1R21* patient derived and control fibroblasts showed increase of p62 in the perinuclear region in patient derived but not in control cells under basal conditions (white arrows). After treatment of cells with MG132, increase of p62-immunoreactivity is detectable at similar level in fibroblasts derived from controls and the *PPP1R21* patient. Scale bars: 20 µm. **D** Studies of cellular fitness by MTT-assay revealed mild reduction of cellular viability mirrored by the metabolic state and membrane permeability in patient-derived cells compared to matched controls. The impact of inhibition of proteasomal activity on cellular viability by MG132 treatment was more pronounced in patient-derived cells compared to controls. **E** A more pronounced increase of cytotoxicity after MG132-treatment is present in patient-derived cells compared to control fibroblasts

The rationale to further focus on proteasome function in the context of PPP1R21opathy is prompted by the fact that impaired vesicular transport may cause protein accumulation and aggregation with a concomitant loss of proteostasis. This in turn often contributes to neurodegenerative diseases, and the ubiquitin–proteasome system plays a major role in protein degradation and proteostasis [21]. Indeed, our proteomic findings displayed an increase of proteins belonging to the ubiquitin–proteasome system (Suppl. Tab. 1) and thus proteasomal activity was analyzed in patient-derived and matched control fibroblasts. A profound increase (fivefold) was observed in *PPP1R21*-mutant cells compared to controls (Fig. 4A). MG132-exposition of control fibroblasts resulted in inhibition of proteasomal activity, and this effect was not observed in patient-derived cells (Fig. 4A).

### Presence of Protein Aggregates in *PPP1R21* Patient-Derived Fibroblasts

Based on previous reports highlighting a role of PPP1R21 in endosomal sorting processes and endosome maturation [4] and the known role of endosomes in protein clearance [22] as well as the increase of proteins involved in the ubiquitin–proteasome system (see above), we studied the presence of protein aggregates by electron microscopy. Our ultra-structural studies revealed a statistically significant increase of myelin-like bodies in cells lacking expression of functional PPP1R21 compared to matched control fibroblasts (Fig. 4B). To confirm our electron microscopic findings, we next studied the presence of protein aggregates by immunolabeling of p62, a known protein aggregate marker. These immunofluorescence-based studies revealed an increased p62-immunoreactivity with protein enrichment within the perinuclear region compared to control fibroblasts in which p62 shows a dot-like distribution within the cytoplasm (Fig. 4C). However, after treatment with MG132, no differences were noticed between control and patient-derived fibroblasts (Fig. 4C).

### Cellular Viability in *PPP1R21* Patient-Derived Fibroblasts

An MTT-assay was performed to investigate the effect of loss of functional PPP1R21 on cellular fitness in presence and absence of the proteasome inhibitor MG132. Our investigation revealed a mild reduction of cellular viability mirrored by the metabolic state and membrane permeability in patient-derived cells compared to matched controls (Fig. 4D). However, the effect of inhibition of proteasomal activity on cellular viability was more pronounced in cells derived from the patient compared to controls. In line with this observation, an increased cytotoxicity after MG132-treatment was identified in patient-derived cells compared to control fibroblasts (Fig. 4E).

### Transcript Studies on RNA Extracted from *PPP1R21* Patient-Derived Fibroblasts

**Fig. 5:**
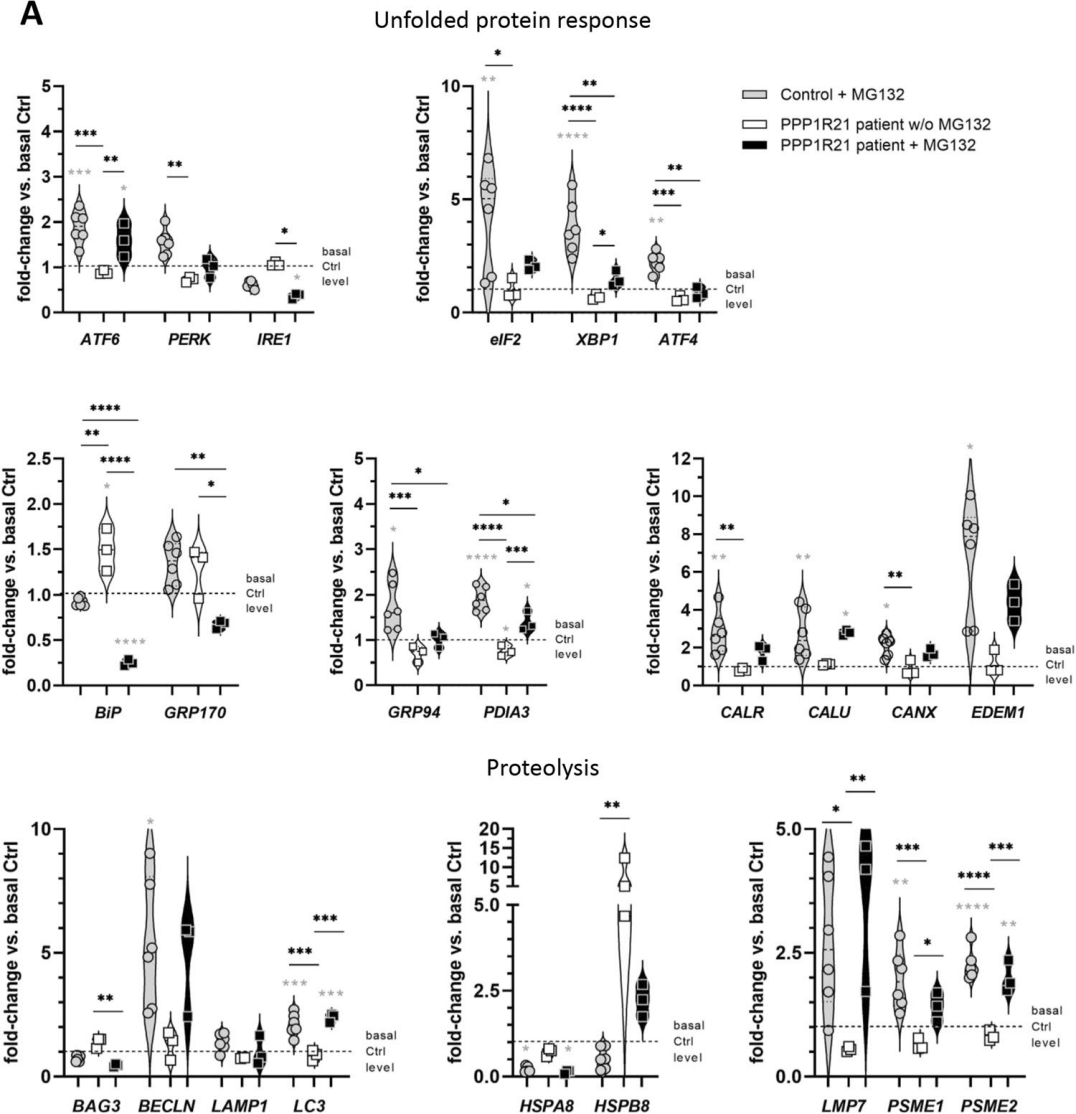

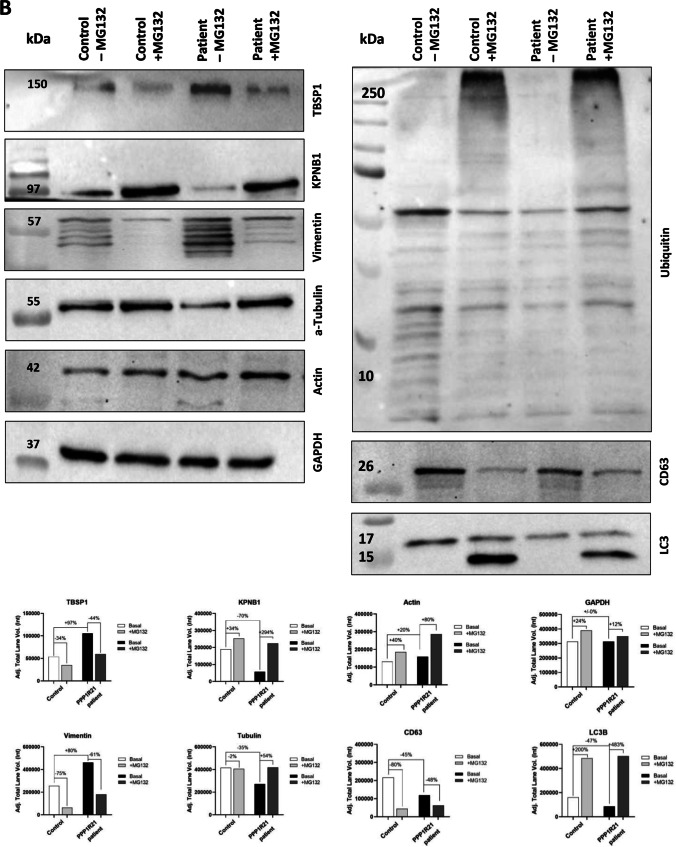
Transcript and immunoblot studies. **A** Quantitative PCR studies of selected transcripts for UPR-modulators (*ATF4*, *ATF6*, *BiP*, *CALR*, *CALU*, *CANX*, *EDEM1*, *EIF2A*, *GRP94*, *GRP170*, *IRE1*, *PDIA3*, *PERK*, and *XBP1*) and proteolysis (*BAG3*, *BECN1*, *HSPA8*, *HSPB8*, *LAMP1*, *LC3*, *LMP7*, *PSME1*, and *PSME2*) revealed a clear effect of MG132 on expression levels of UPR-related genes in control fibroblast. Furthermore, PPP1R21 patient and control fibroblasts present with comparable expression level under basal control expression, with exception of *BiP* and *GRP170*. Treatment with MG132 decreased the expression of both UPR-modulators, while other markers showed elevated expression. *PERK*, *XBP1*, and *GRP94* only show increase upon MG132 treatment in control but not patient-derived fibroblasts. Among autophagy modulating factors, *BECLN*, *LC3*, and *HSPB8* as well as the transcripts *LMP7*, *PSME*1, and *PSME2* encoding for proteasomal proteins were increased upon MG132 treatment in both, patient derived and control fibroblasts. None of the autophagy modulating factors or proteasomal protein show an increase under basal conditions in patient-derived fibroblasts. **B** Immunoblot studies (including quantification) focusing on proteins belonging to cytoskeleton (alpha-tubulin, vimentin, and actin), KPNB1 as being identified to be dysregulated by proteomic profiling, and such involved in protein clearance (ubiquitin, CD63, and LC3) confirmed affection of cytoskeleton and protein degradation in fibroblasts lacking expression of PPP1R21 and moreover show alterations of these processes in cells treated with MG132 by the detection of increase of vimentin, actin, and ubiquitin and reduced conversion from LC3-I to LC3-II

Quantitative PCR studies of selected transcripts covering transcripts encoding for proteins modulating the unfolded protein response (major transducers: *ATF6*, *IRE1*, and *PERK*; direct related down-stream factors: *ATF4*, *XBP1*, and *EIF2A*; indirect related down-stream factors: *BiP* (major ER-chaperone) along with its co-chaperone GRP170 and further UPR-modulated proteins *CALR*, *CALU*, *CANX*, *EDEM1*, *GRP94*, *PDIA2*, *PDIA3*, and *SEC63*) and proteolysis (direct modulators of autophagy: *BAG3*, *BECN1*, *LAMP1*, and *LC3*; autophagy assisting chaperones: *HSPA8* and *HSPB8*; proteasome factors: *LMP7*, *PSME1*, and *PSME2*) were performed. These studies revealed a clear effect of MG132 on expression levels of UPR-related genes in control fibroblasts, shown by significant increased expression of multiple genes after treatment (Fig. 5A, e.g., *ATF6*, *EIF2* or *GRP94*, *CALR*, and *CALU*). Additionally, we demonstrated that PPP1R21 patient fibroblasts showed a comparable expression level to basal control level without treatment, except for *BiP* and *GRP170*, which were significantly increased when compared to basal control levels. Treatment with MG132 decreased the expression of both transcripts of these two UPR factors, while other markers showed elevated levels after treatment (Fig. 5A, e.g., *ATF6*, *XBP1*, *PDIA3*, or *EDEM1*). Activated autophagy after blocking of the proteasome by MG132 treatment is indicated by statistically significant increased expression of *LC3* to similar extend in controls and patient-derived cells as well as by comparable increase of *BECLN* (not statistically significant in patient-derived cells). *BAG3* is decreased in patient derived but not in control fibroblasts after MG132 treatment. Proteasomal genes *LMP7*, *PSME1*, and *PSME2* were highly increased after treatment in control, as well as patient fibroblasts but surprisingly not under basal conditions in patient-derived cells (Fig. 5A).

### Immunoblot Studies on Protein Extracts of *PPP1R21* Patient-Derived Fibroblasts

To verify our proteomic findings and to further focus on the impact of an activated protein clearance machinery and on the dysregulation of cytoskeletal proteins, immunoblot studies were carried out on whole protein extracts of fibroblasts derived from our *PPP1R21* patient as well as from two matching pooled controls (basal conditions and after MG132-treatment). This showed lower level of total ubiquitinated proteins under basal conditions in whole proteins extracts of fibroblasts derived from controls compared to the index patient, a finding which accords with the increased proteasomal activity in patient cells. After MG132-treatment, level of total ubiquitinated proteins is increased in both, patient-derived and control fibroblasts (Fig. 5B). Under basal conditions, LC3-I and CD63 level are lower in patient-derived fibroblasts. Moreover, less conversion of LC3-I to LC3-II is observed after MG132 treatment in patient-derived fibroblasts compared to pooled controls (Fig. 5B). Focusing on cytoskeletal proteins, vimentin and actin are increased in patient-derived fibroblasts under basal conditions as well as after treatment with MG132 compared to pooled control cells (Fig. 5B). In contrast, alpha-tubulin shows decreased level under basal conditions but not after MG132-treatment in patient-derived fibroblasts compared to controls (Fig. 5B). In line with our proteomic findings, KBNP1 is decreased in *PPP1R21*-mutant fibroblasts under basal conditions, but no differences were noticed between patient-derived and control fibroblasts after MG132-treatment (Fig. 5B).

### Actin Cytoskeleton Rearrangement in *PPP1R21* Patient-Derived Fibroblasts Treated with MG132

**Fig. 6:**
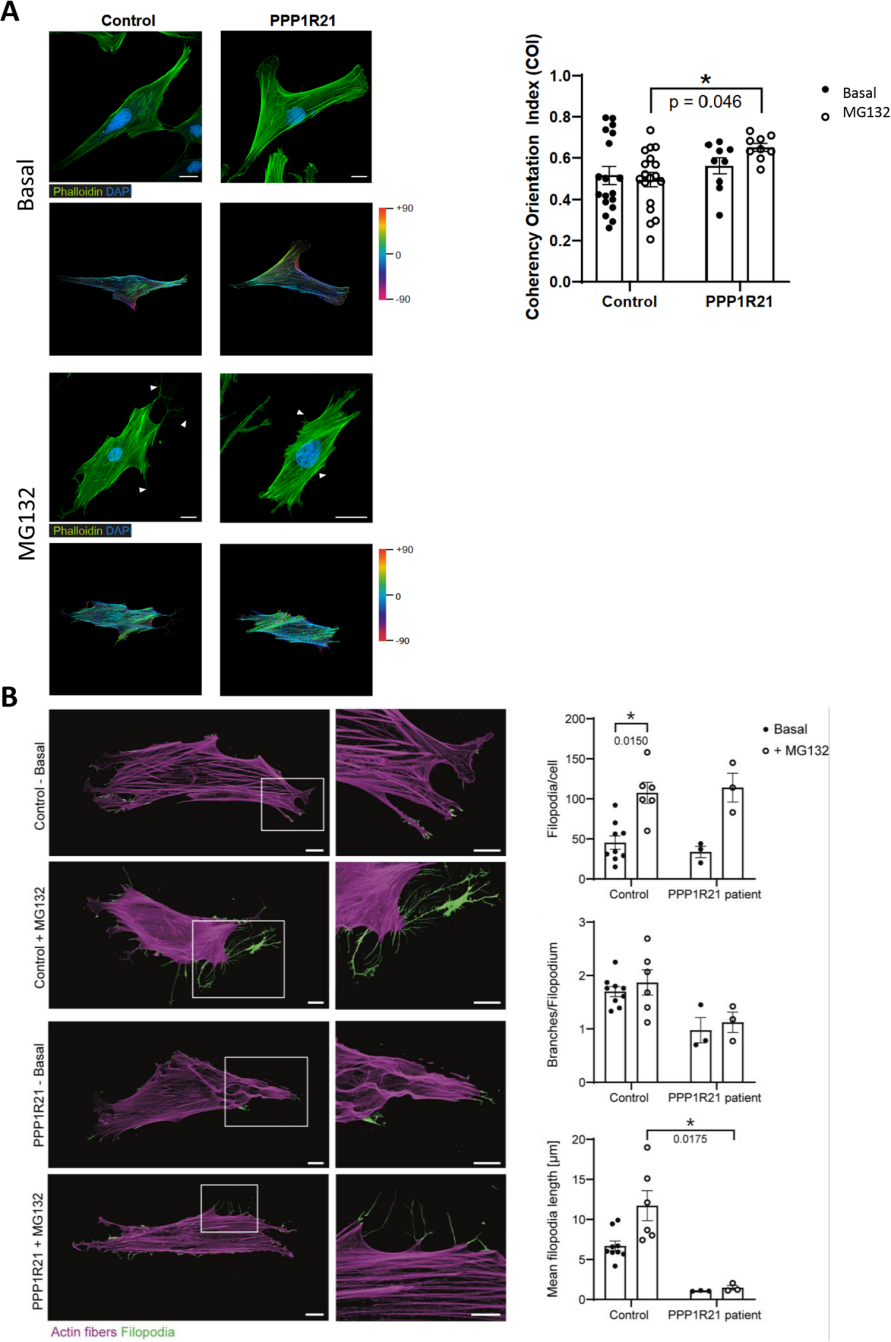
Studies of actin cytoskeleton. **A** Actin filaments (Phalloidin, green) and nuclei (DAPI, blue) of control and PPP1R21 patient-derived fibroblasts are visualized via confocal laser scanning microscopy. MG132 treated fibroblasts of control patients exhibit longer and more branched filopodia (white arrows) than PPP1R21 patient fibroblasts. Scale bars: 20 µm. Circular color map coding displays the local actin filament orientation of the basal and MG132 treated fibroblasts derived from controls and the PPP1R21 patient, respectively. The color gradient indicates the spatial orientation of the actin fibers from − 90° to + 90°. MG132 treatment induced a significant increase in the cytoskeletal coherency of PPP1R21 patient fibroblasts compared to stimulated controls. **B** Microscopic studies of fibroblast filopodia (illustrated in green) revealed an effect of MG132 exposure in terms of an increase of filopodia formation per cell in both, control- and patient-derived cells. Branches and length per filopodia are (slightly) reduced in patient-derived cells and. Mean filopodia length increased upon MG132 treatment only in control fibroblasts. Scale bars in overview images = 20 µm, scale bars in magnification = 10 µm

Prompted by our proteomic findings showing an impact of loss of functional PPP1R21 on the abundance of cytoskeletal proteins, FITC-phalloidin staining was carried out on control and patient-derived fibroblasts under basal conditions and after MG132-treatment to study actin-architecture. Calculation of the coherency orientation index (COI) in 18 control fibroblasts under basal conditions revealed an index comparable to the ones in patient-derived fibroblasts (0.516 and 0.561). After treatment of cells with MG132, no particular change was noticed in the control fibroblasts (0.516 versus 0.496) whereas in patient-derived cells, a slight increase was detected (0.561 versus 0.651). MG132-treated *PPP1R21* patient-derived fibroblasts show a significantly increased COI compared with MG132-treated control fibroblasts (0.651 versus 0.496), indicating impaired actin remodeling in *PPP1R21*-mutant fibroblasts (Fig. 6A). Given that filopodia represent thin, actin-rich plasma-membrane protrusions that function as antennae for cells, we also studied these cellular structures by confocal imaging. Indeed, these microscopic studies revealed a significant increase in filopodia number per cell after MG132-treatment in the healthy control fibroblasts and the same trend in the *PPP1R21* patient-derived fibroblasts. Moreover, in both, fibroblasts derived from healthy controls and the *PPP1R21* patient, MG132-treatment tends to increase the branching and length of filopodia. Furthermore, MG132-treament results in a notable difference in filopodia length between the healthy control fibroblasts and the fibroblasts derived from the *PPP1R21* patient (Fig. 6B).

## Discussion

Here, we report on the first *PPP1R21* patient, harboring a homozygous splice variant (c.748-3A > G), providing associated phenotypical data and clinical comparisons with previously reported *PPP1R21* patients. We also describe novel pathophysiological insights driven by the findings of our proteomic profiling on patient-derived fibroblasts.

The phosphatase 1 regulatory subunit 21 protein is encoded by the *PPP1R21* gene (OMIM*618,159) and belongs to a family of coiled-coil domain containing proteins, an evolutionarily conserved class of proteins with important roles in several developmental processes. Over the last three years, homozygous variants in *PPP1R21* have been described in 11 patients, most of them sharing developmental delay/intellectual disability, hypotonia, distinctive facial features, and brain anomalies [7]. In consistency with the previous reports [4–8], our patient showed a broad spectrum of clinical signs. He presented with respiratory distress shortly after birth (8/11), hypotonia (10/11), distinct developmental delay, and ID (9/11), including absent speech at the age of six. He showed some of the dysmorphisms described previously, e.g., coarse features (8/11), very low set ears (7/11), skull asymmetry (4/11), long philtrum (2/11), and a small nasal bridge (5/11). Additional signs were strabismus (5/11) and cryptorchidism (2/11). Other frequent signs like feeding problems (8/11), cardiac abnormalities (5/11), or scoliosis (5/11) were not present in our patient. CNS anomalies, e.g., thin corpus callosum (6/11), cerebellar vermis hypoplasia (5/11), or reduced white matter (5/11) were not identified in our patient. However, an initial MRI showed small bleeding foci in the right central region and the follow-up showed a moderate PVL. Overall, the clinical presentation of our patient is milder than the patients described so far.

In line with the known functions of PP1 as an important serine/threonine phosphatase essential for glycogen metabolism [2], altered sugar metabolism is indicated in our in vitro model lacking expression of functional PPP1R21 (see proteomic findings). In addition, a role of PP1 in protein synthesis is known [23] and results of our proteomic profiling on patient-derived fibroblasts indicate an impact on protein production on the RNA level. Notably, PP1 is known to be expressed during the development in different neuronal cytoskeletal compartments [3] and also known to play a role in muscle contractility despite many other processes. Proper cytoskeleton is crucial for neuronal plasticity and function as well as vesicular transport processes. Altered vesicular transport was recently linked to the pathophysiology based on loss of functional PPP1R21 [4].

Prompted by our proteomic findings indicative for activation of the ubiquitin–proteasome system, functional, transcriptional, and immunoblot studies were performed to investigate proteolysis in *PPP1R21*-mutant and control fibroblasts. These studies revealed a pronounced activation of the proteasome, as a multi-catalytic protease that degrades cellular proteins, even under treatment with MG132 in patient-derived cells. This finding is in line with our proteomic results showing a significant increase of a variety of proteins involved in the function of this protein clearance system as well as with the decrease of ubiquitinated proteins and LC3-I along with CD63 (two proteins involved in autophagy-related protein clearance) under basal conditions. Along this line, activation of the proteasome might reflect a cellular defense mechanism antagonizing the (massive) cytotoxic build-up of protein aggregates which have been identified with increased abundance in patient-derived fibroblasts by electron microscopy and immunofluorescence studies of p62. Hence, the co-occurrence of considerable elevated proteasomal activity and protein aggregates in fibroblasts of the PPP1R21-patient under basal conditions most likely correlates in terms of a cellular attempt to elevate protein clearance capacity to facilitate break-down of protein aggregates. Hereby, results of our combined transcript and proteomic studies indicate that activation of the proteasome in patient-derived fibroblasts is not related to an increased expression of proteasomal proteins (transcript level remain unchanged) and that their increased abundance might rather arise from altered protein stability toward forced proteasomal-based protein clearance. Results of our transcript studies revealed increased level of *BiP* and *GRP170* encoding for a crucial chaperone system known to function as an analogous protein-disaggregation system activated under stress conditions in terms of a proteostasis-restorative ER-stress response [24]. Chaperone-assisted protein-disaggregation under basal conditions is moreover suggested by increase of *HSPB8*. However, further proteomic data are not indicative for a broad activation of the ER-stress response, thus confirming the concept of protein aggregation arising beyond the protein production (within the ER). The UPS serves as the major protein quality control system in the eukaryotic cells, responsible for approximately 80–90% of the cellular protein degradation [25]. This prompted us to further elucidate the impact of this protein clearance machinery in der underlying pathophysiology. For that purpose, we used MG132, one of the most widely used proteasome inhibitors in basic research. This inhibitor binds to the β5 β1 and β2 subunits of the 20S proteasome [26]. Interestingly, results of our studies on MG132-treated control and patient-derived fibroblasts showed an inhibitory effect only in control cells. One might speculate that this might reflect an activation of proteasomal activity in proteasomal subunits or regulators beyond those targeted by MG132 (for instance, ECM29, PSMF, PA28αβ, PA28γ, and PA200 complexes [27]) in PPP1R21opathy. The proteomic signature of fibroblasts derived from our PPP1R21-patient shows a dysregulation of several proteins supporting the assumption of a direct impact of loss of functional PPP1R21 on physiological proteasome function. These proteins include the proteasome assembly chaperones 2 and 4, the proteasome activator activator complex subunit 2, the 26S proteasome non-ATPase regulatory subunits 7 and 10, and the E3 ubiquitin-protein ligase HECTD3, an enzyme implicated in the ubiquitylation of a variety of proteasomal subunits as a post-translational modification having a positive impact in proteasome activity [28]. Further functional studies utilizing inhibitors targeting these subunits or particular regulating proteins are needed to proof the assumption of a (direct) impact of PPP1R21 abundance on physiological proteasome function.

Notably, our MTT-assay revealed decreased cellular proliferation, an observation in agreement with the known function of PP1 in cell cycle regulation, accompanied with increased cellular toxicity burden. Moreover, a crosstalk between proteasome-mediated protein degradation and cytoskeleton has been described in the context of different studies [29] including those focusing on motoneuron differentiation [30]. Our proteomic findings and immunoblot data also indicate a direct impact of the loss of functional PPP1R21 on cytoskeleton in fibroblasts by the dysregulation of various proteins playing crucial roles in proper cytoskeleton. Although no alteration of actin-cytoskeleton-architecture was noticed by comparing immunofluorescence findings of control and patient-derived cells under basal conditions, we observed altered orientation (increase of the coherency orientation index) of actin bundles and altered formation of filopodia in patient-derived fibroblasts treated with MG132, a known inhibitor of the proteasome and activator of autophagy (as shown by our immunoblot findings) compared to treated control cells. In this context, although *LC3* transcript level is increased, our immunoblot studies showed a reduced conversion of LC3-I to LC3-II in patient-derived cells treated with MG132 thus suggesting a partial impairment of autophagy-activation. This finding is supported by decrease of *BAG3* in patient derived but not in control fibroblasts after MG132 treatment. Hence, our combined data suggest an impact of the activated protein clearance machinery on proper cytoskeleton which might impact on the regular transport of vesicles. However, further functional studies are needed to precisely decipher this molecular interplay in the context of PPP1R21 etiopathology.

Apart from indicating an activation of the ubiquitin–proteasome system, our proteomic study unraveled the dysregulation of various proteins known to play essential roles along the neuromuscular axis such as ataxin-10 and sacsin (SACS), both are linked to the manifestation of ataxia and cerebellar integrity. As mentioned above, our patient did not present with cerebellar abnormalities as observed in other *PPP1R21*-patients. Given that expression level of sacsin was already linked to proper cytoskeleton and proteolysis [31], one might speculate that increased abundance in *PPP1R21*-mutant fibroblasts reflects the activation of cellular mechanisms to antagonize pathophysiological cascades initiated by the loss of functional PPP1R21. Increased abundance of supervillin might reflect the same hypothesis as loss of expression of functional supervillin was linked to the manifestation of myopathy characterized by myofibrillar disorganization and build-up of protein aggregates. Another interesting candidate in this context is neurabin-2: this protein also belongs to the PPP1R-protein family (PPP1R9B), modulates excitatory synaptic transmission and dendritic spine morphology, and operates in cross-lining of F-actin [32]. However, further functional studies (on neurons) are needed to molecularly dissect the impact of the individual proteins as protecting factors or modifiers in the etiopathology of PPP1R21 deficiency. Along this line, proteomic profiling also unraveled the dysregulation of various proteins involved in metabolic processes. Thus, it is very likely that altered metabolism also contributes of the biochemical etiology of PPP1R21opathy in terms of a multi-pathophysiological incident.

Although this study highlights the power of proteomics as an emerging tool for analysis of consequences of genetic variants even in individual patients suffering from nano-rare diseases, confirmational studies on fibroblasts derived from further *PPP1R21*-patients are needed to generalize our findings and the related conclusions. Moreover, confirmational studies on cellular populations clearly vulnerable in PPP1R21opathy such as neurons are crucial to investigate the role of the neuronal relevant proteins in the molecular etiology of the disease. Organoids or animal models would be needed for this purpose. Further studies on other perturbed cellular processes such as metabolism are needed to address the overall pathophysiology of PPP1R21opathy.

## Conclusions

In conclusion, our study extended the molecular genetic spectrum of PPP1R21opathy by describing the first splice variant leading to loss of protein expression and hereby described a patient presenting with a milder phenotype of NEDHFBA. Proteomic studies unraveled a dysregulation of various proteins playing crucial roles in proper function of the neuromuscular axis and increase of a variety of such might reflect the activation of cellular defense mechanisms. Our combined biochemical, microscopic, and cell biological investigations revealed increased presence of protein aggregates along with elevated cytotoxicity and activation of ubiquitin–proteasome system as well as altered cytoskeleton, a molecular crosstalk already described in the context of other diseases [29]. Latter one is also influenced by modulation of proteolysis and provides new insights into the pathophysiology of PPP1R21opathy. Finally, our data confirm the concept that human fibroblasts may serve as a suitable in vitro model to study the molecular etiology of rare neurological diseases [30].

## Supplementary Information

Below is the link to the electronic supplementary material.

Supplementary file1 Dysregulated proteins their localizations, functions and associated diseases (XLSX 15 KB)

Supplementary file2 Antibodies used in this study (DOCX 14 KB)

## Data Availability

The mass spectrometry proteomics data have been deposited to the ProteomeXchange Consortium via the PRIDE [26] partner repository with the dataset identifier PXD034993. For reviewers: Username: reviewer_pxd034993@ebi.ac.uk. Password: h2GP3aQV.

## References

[1] Peel N, Iyer J, Naik A (2017). Protein phosphatase 1 down regulates ZYG-1 levels to limit centriole duplication. PLoS Genet.

[2] Munro S, Cuthbertson DJR, Cunningham J (2002). Human skeletal muscle expresses a glycogen-targeting subunit of PP1 that is identical to the insulin-sensitive glycogen-targeting subunit G(L) of liver. Diabetes.

[3] Strack S, Kini S, Ebner FF (1999). Differential cellular and subcellular localization of protein phosphatase 1 isoforms in brain. J Comp Neurol.

[4] Rehman AU, Najafi M, Kambouris M (2019). Biallelic loss of function variants in PPP1R21 cause a neurodevelopmental syndrome with impaired endocytic function. Hum Mutat.

[5] Anazi S, Maddirevula S, Salpietro V (2017). Expanding the genetic heterogeneity of intellectual disability. Hum Genet.

[6] Kotecha UH, Mistri M, Rayabarapu P (2022). The diagnostic utility of exome-based carrier screening in families with a positive family history. Am J Med Genet A.

[7] Loddo S, Alesi V, Radio FC (2020). PPP1R21-related syndromic intellectual disability: report of an adult patient and review. Am J Med Genet A.

[8] Suleiman J, Al Hashem AM, Tabarki B (2018). PPP1R21 homozygous null variants associated with developmental delay, muscle weakness, distinctive facial features, and brain abnormalities. Clin Genet.

[9] Roos A, Thompson R, Horvath R et al (2018) Intersection of proteomics and genomics to “solve the unsolved” in rare disorders such as neurodegenerative and neuromuscular diseases. Proteomics Clin Appl. 10.1002/prca.201700073 10.1002/prca.201700073 29059504

[10] Burkhart JM, Schumbrutzki C, Wortelkamp S (2012). Systematic and quantitative comparison of digest efficiency and specificity reveals the impact of trypsin quality on MS-based proteomics. J Proteomics.

[11] Hentschel A, Ahrends R (2020). Developing a robust assay to monitor and quantify key players of metabolic pathways during adipogenesis by targeted proteomics. Proteomics.

[12] Liebermeister W, Noor E, Flamholz A (2014). Visual account of protein investment in cellular functions. Proc Natl Acad Sci U S A.

[13] Snel B, Lehmann G, Bork P (2000). STRING: a web-server to retrieve and display the repeatedly occurring neighbourhood of a gene. Nucleic Acids Res.

[14] Guettsches A-K, Meyer N, Zahedi RP et al (2022) FYCO1 increase and effect of arimoclomol-treatment in human VCP-pathology. Biomedicines 10(10):2443. 10.3390/biomedicines10102443 10.3390/biomedicines10102443 PMC9598455 36289705

[15] Arlt A, Kohlschmidt N, Hentschel A (2022). Novel insights into PORCN mutations, associated phenotypes and pathophysiological aspects. Orphanet J Rare Dis.

[16] Arganda-Carreras I, Fernández-González R, Muñoz-Barrutia A (2010). 3D reconstruction of histological sections: application to mammary gland tissue. Microsc Res Tech.

[17] Voigt M, Fusch C, Olbertz D (2006). Analyse des Neugeborenenkollektivs der Bundesrepublik Deutschland. Geburtsh Frauenheilk.

[18] Kromeyer-Hauschild K, Wabitsch M, Kunze D (2001). Perzentile für den body-mass-index für das Kindes- und Jugendalter unter Heranziehung verschiedener deutscher Stichproben. Monatsschr Kinderheilkd.

[19] Braegger C, Jenni OG, Konrad D (2011). Neue Wachstumskurven für die Schweiz.

[20] Hentschel A, Czech A, Münchberg U (2021). Protein signature of human skin fibroblasts allows the study of the molecular etiology of rare neurological diseases. Orphanet J Rare Dis.

[21] Thibaudeau TA, Anderson RT, Smith DM (2018). A common mechanism of proteasome impairment by neurodegenerative disease-associated oligomers. Nat Commun.

[22] Buratta S, Tancini B, Sagini K et al (2020) Lysosomal exocytosis, exosome release and secretory autophagy: the autophagic- and endo-lysosomal systems go extracellular. Int J Mol Sci 21(7):2576. 10.3390/ijms21072576 10.3390/ijms21072576 PMC7178086 32276321

[23] Maltsev AV, Balaban PM (2021). PP1/PP2A phosphatase inhibition-induced metaplasticity in protein synthesis blocker-treated hippocampal slices: LTP and LTD, or there and back again. Biochem Biophys Res Commun.

[24] Melo EP, Konno T, Farace I (2022). Stress-induced protein disaggregation in the endoplasmic reticulum catalysed by BiP. Nat Commun.

[25] Kwon YT, Ciechanover A (2017). The ubiquitin code in the ubiquitin-proteasome system and autophagy. Trends Biochem Sci.

[26] Kisselev AF, Goldberg AL (2001). Proteasome inhibitors: from research tools to drug candidates. Chem Biol.

[27] Albornoz N, Bustamante H, Soza A et al (2019) Cellular responses to proteasome inhibition: molecular mechanisms and beyond. Int J Mol Sci 20(14):3379. 10.3390/ijms20143379 10.3390/ijms20143379 PMC6678303 31295808

[28] Martinez A, Ramirez J, Osinalde N (2018). Neuronal proteomic analysis of the ubiquitinated substrates of the disease-linked E3 ligases Parkin and Ube3a. Biomed Res Int.

[29] Ibañez-Vega J, Del Valle BF, Saez JJ (2019). Proteasome dependent actin remodeling facilitates antigen extraction at the immune synapse of B cells. Front Immunol.

[30] Bax M, McKenna J, Do-Ha D et al (2019) The ubiquitin proteasome system is a key regulator of pluripotent stem cell survival and motor neuron differentiation. Cells 8(6):581. 10.3390/cells8060581 10.3390/cells8060581 PMC6627164 31200561

[31] Duncan EJ, Larivière R, Bradshaw TY (2017). Altered organization of the intermediate filament cytoskeleton and relocalization of proteostasis modulators in cells lacking the ataxia protein sacsin. Hum Mol Genet.

[32] Sagara M, Kawasaki Y, Iemura S (2009). Asef2 and Neurabin2 cooperatively regulate actin cytoskeletal organization and are involved in HGF-induced cell migration. Oncogene.

