# Correction: A Homozygous PPP1R21 Splice Variant Associated with Severe Developmental Delay, Absence of Speech, and Muscle Weakness Leads to Activated Proteasome Function

**DOI:** 10.1007/s12035-023-03319-6

**Published:** 2023-03-21

**Authors:** Andreas Hentschel, Nancy Meyer, Nicolai Kohlschmidt, Claudia Groß, Albert Sickmann, Ulrike Schara-Schmidt, Fabian Förster, Ana Töpf, Jon Christiansen, Rita Horvath, Matthias Vorgerd, Rachel Thompson, Kiran Polavarapu, Hanns Lochmüller, Corinna Preusse, Luis Hannappel, Anne Schänzer, Anika Grüneboom, Andrea Gangfuß, Andreas Roos

**Affiliations:** 1 Leibniz-Institut für Analytische Wissenschaften – ISAS – e.V, Dortmund, Germany; 2 Department of Pediatric Neurology, Centre for Neuromuscular Disorders, Centre for Translational Neuro- and Behavioral Sciences, University Duisburg-Essen, Essen, Germany; 3 Institute of Clinical Genetics and Tumor Genetics, Bonn, Germany; 4 The John Walton Muscular Dystrophy Research Centre, Translational and Clinical Research Institute, Newcastle University and Newcastle Hospitals NHS Foundation Trust, Newcastle Upon Tyne, UK; 5 Department of Clinical Neurosciences, John Van Geest Centre for Brain Repair, School of Clinical Medicine, University of Cambridge, Cambridge, UK; 6 Department of Neurology, Heimer Institute for Muscle Research, University Hospital Bergmannsheil, Ruhr University Bochum, Bochum, Germany; 7 Children’s Hospital of Eastern Ontario Research Institute, Division of Neurology, Department of Medicine, The Ottawa Hospital; and Brain and Mind Research Institute, University of Ottawa, Ottawa, Canada; 8 Department of Neuropathology, Charité - Universitätsmedizin Berlin, Berlin, Germany; 9 Institute of Neuropathology, Justus Liebig University, Gießen, Germany

**Correction: Molecular Neurobiology**

**https://doi.org/10.1007/s12035-023-03219-9**

The original version of this article unfortunately contained mistake. One of the author name was missspelled during revision and finalization of the manuscript.

Dr. Kiran Polavarapu's surname "Polaparapu" should be spelled "Polavarapu".

The original article has been corrected.

