# Supplementary material for: A Homozygous PPP1R21 Splice Variant Associated with Severe Developmental Delay, Absence of Speech, and Muscle Weakness Leads to Activated Proteasome Function
Source: Mol Neurobiol. 2023 Jan 24;60(5):2602–18. doi: 10.1007/s12035-023-03219-9 (PMC10039818; doi:10.1007/s12035-023-03219-9)
Supplement: Supplementary file 2 — Supplementary file2 Antibodies used in this study (DOCX 14 KB) [file 12035_2023_3219_MOESM2_ESM.docx]

| **Antibody** | **Supplier** | **Dilution** |
| --- | --- | --- |
| PPP1R21 (antibody 1 in figure 2C) | Bethyl (A303-824) | 1:500 (immunoblotting) |
| PPP1R21 (antibody 2 in figure 2C) | Thermo Fisher (#PA5-57820) | 1:500 (immunoblotting) |
| P62/ SQSTM1 | Abcam (ab109012) | 1:100 (immunofluorescence) |
| TBSP1/Thrombospondin-1 | Abcam (ab85762) | 1:1000 (immunoblotting) |
| Importin subunit beta-1/ KPNB1 | Abcam (ab2811) | 1:1000 (immunoblotting) |
| Vimentin | Genetex (GTX112661) | 1:750 (immunoblotting) |
| Alpha-Tubulin | Cell Signaling (#2144) | 1:1000 (immunoblotting) |
| Actin | Santa Cruz (sc-47778) | 1:1000 (immunoblotting) |
| GAPDH | Genetex (GTX108711) | 1:1000 (immunoblotting) |
| Ubiquitin | Abcam (ab19247) | 1:1000 (immunoblotting) |
| CD63 | Abcam (ab8219) | 1:500 (immunoblotting) |
| LC3 | Abcam (ab51520) | 1:500 (immunoblotting) |
